# The antipsychotic drugs olanzapine and haloperidol modify network connectivity and spontaneous activity of neural networks *in vitro*

**DOI:** 10.1038/s41598-017-11944-0

**Published:** 2017-09-14

**Authors:** Egor Dzyubenko, Georg Juckel, Andreas Faissner

**Affiliations:** 1 Department of Cell Morphology and Molecular Neurobiology, Faculty of Biology and Biotechnology, Ruhr University Bochum, Universitaetsstr. 150, Ruhr-University, building NDEF 05, D-44801 Bochum, Germany; 2 LWL University Hospital Department of Psychiatry, Psychotherapy and Preventive Medicine, Ruhr University Bochum, Alexandrinenstrasse 1-3, D-44791 Bochum, Germany

## Abstract

Impaired neural synchronization is a hallmark of psychotic conditions such as schizophrenia. It has been proposed that schizophrenia-related cognitive deficits are caused by an unbalance of reciprocal inhibitory and stimulatory signaling. This supposedly leads to decreased power of induced gamma oscillations during the performance of cognitive tasks. In light of this hypothesis an efficient antipsychotic treatment should modify the connectivity and synchronization of local neural circuits. To address this issue, we investigated a model of hippocampal neuronal networks *in vitro*. Inhibitory and excitatory innervation of GABAergic and glutamatergic neurons was quantified using immunocytochemical markers and an automated routine to estimate network connectivity. The first generation (FGA) and second generation (SGA) antipsychotic drugs haloperidol and olanzapine, respectively, differentially modified the density of synaptic inputs. Based on the observed synapse density modifications, we developed a computational model that reliably predicted distinct changes in network activity patterns. The results of computational modeling were confirmed by spontaneous network activity measurements using the multiple electrode array (MEA) technique. When the cultures were treated with olanzapine, overall activity and synchronization were increased, whereas haloperidol had the opposite effect. We conclude that FGAs and SGAs differentially affect the balance between inhibition and excitation in hippocampal networks.

## Introduction

Psychotic disorders including schizophrenia are severe diseases which affect up to 1% of the population, causing a heavy economic burden on society^1^. Therefore, the in-depth understanding of the underlying pathophysiology is an important objective of psychiatric research. Schizophrenia is characterized by a sequence of acute and stable phases switching from the prevalence of positive to negative symptoms, accompanied with cognitive impairment and depression^2^. Throughout these phases, the antipsychotic medications are chronically administered to control a patient’s symptoms and to improve life quality. The dopamine hypothesis proposed that the preponderance of dopaminergic activity is central for the etiology of psychosis and dopamine receptors antagonists such as the first generation antipsychotic (FGA) haloperidol were used as primary treatments^3^. Nowadays, the judicious regulation of dopamine, serotonin, and histamine receptors is intended, and second generation antipsychotics (SGA) such as olanzapine are more frequently prescribed^4^.

The recently proposed GABA-glutamate theory of schizophrenia suggests that the inhibition-excitation imbalance within local neural circuits constitutes a central mechanism of the disease^5^. The manifestation of cognitive and learning deficits in psychotic patients can be attributed to the reduced power of gamma oscillations^6^. This dysfunction arises in part from a compromised excitation-inhibition balance and impaired synchronization within cortical and hippocampal neural networks^7–9^. In this context, the enhancement of glutamatergic synaptogenesis by atypical antipsychotics^10–12^ may represent an aspect of their mode of action, as they appear to promote the excitatory drive in local circuits. In addition, the antipsychotic treatment partially restored the deficit of adult neurogenesis and neuronal maturation in both rodent models and in the human, the latter according to postmortem tissues^13, 14^, indicating enhanced neural plasticity. Taken together, these studies indicate the potential of antipsychotics to modify the establishment and plasticity of neural networks. However, the balance of the GABA-glutamate circuitry and its impact on functional network states has so far barely been studied.

Inhibitory interneurons provide the essential control of the excitation-inhibition balance and synchronization in local neuronal networks^15^. In schizophrenia, the reduction of parvalbumin-containing interneurons (PV interneurons) density and the compromised formation of perineuronal nets (a special layer of extracellular matrix that regulates synaptic plasticity^16^, for review see refs 17–20), correlate with altered cortical networks oscillations^6^. This evidence motivated us to focus on the individual analysis of inhibitory interneurons and interacting glutamatergic cells using primary cultures of hippocampal neurons.

Different types of neurons undergo distinct steps of maturation both *in vitro* and *in vivo* ^21^. In particular, GABAergic interneurons begin to exert inhibitory activity at relatively later stages of neuronal network maturation, typically after 14–21 days *in vitro* (DIV) in primary cell cultures^22–24^. In our model, the mature state of neuronal networks is reached by approximately 21 DIV^25^. For this reason, three weeks old cultures were examined in most experiments.

In several earlier studies, the in-depth investigation of network synchronization mechanisms was performed with the help of computational approaches^26, 27^. However, very few^28^ studies addressed the effects of drug administration using *in silico* neural networks simulation. On the other hand, pharmacological studies *in vitro* do not provide the understanding of network activity related effects. In the current study, we combined cell type specific synapse quantification, spontaneous network activity measurements and neural network simulations to advance our understanding as to how FGA and SGA medication can modify synaptic density and activity patterns of neural networks.

## Results

### The composition of neuronal networks is not altered by antipsychotics

In order to understand how FGA and SGA exposure modify the functional states of neural networks, we first defined the composition and survival of primary cultures of embryonic hippocampal neurons upon treatment. In mature cultures, both interneuron markers GABA and parvalbumin were co-expressed with aggrecan, indicating that this proteoglycan may serve as a reliable marker of inhibitory interneurons (Supplementary Fig. S1a). After 21 DIV, the cultivated networks comprised approximately 35% GABAergic parvalbumin expressing interneurons. Considering that glutamatergic neurons are the predominant neuron type in the hippocampus, we assume that the remaining 65% are pyramidal excitatory cells. According to the expression of aggrecan, GABA and parvalbumin, the proportion of inhibitory interneurons was not affected by the chronic application of olanzapine or haloperidol (at 100 nM each, Supplementary Fig. S1b). The concentrations of drugs were selected on the basis of their target receptor affinity (reviewed by ref. 13), recommended concentrations in patient’s blood plasma^29^, or preliminary survival tests (Supplementary Fig. S1c,d) and were comparable with the dosages used *in vitro* by other researchers^10, 11^. Our observations suggest that 100 nM haloperidol and 100 nM olanzapine did not compromise the composition and survival of neuronal cultures.

### Olanzapine and haloperidol modify GABA- and glutamatergic synapse density

**Figure 1:**
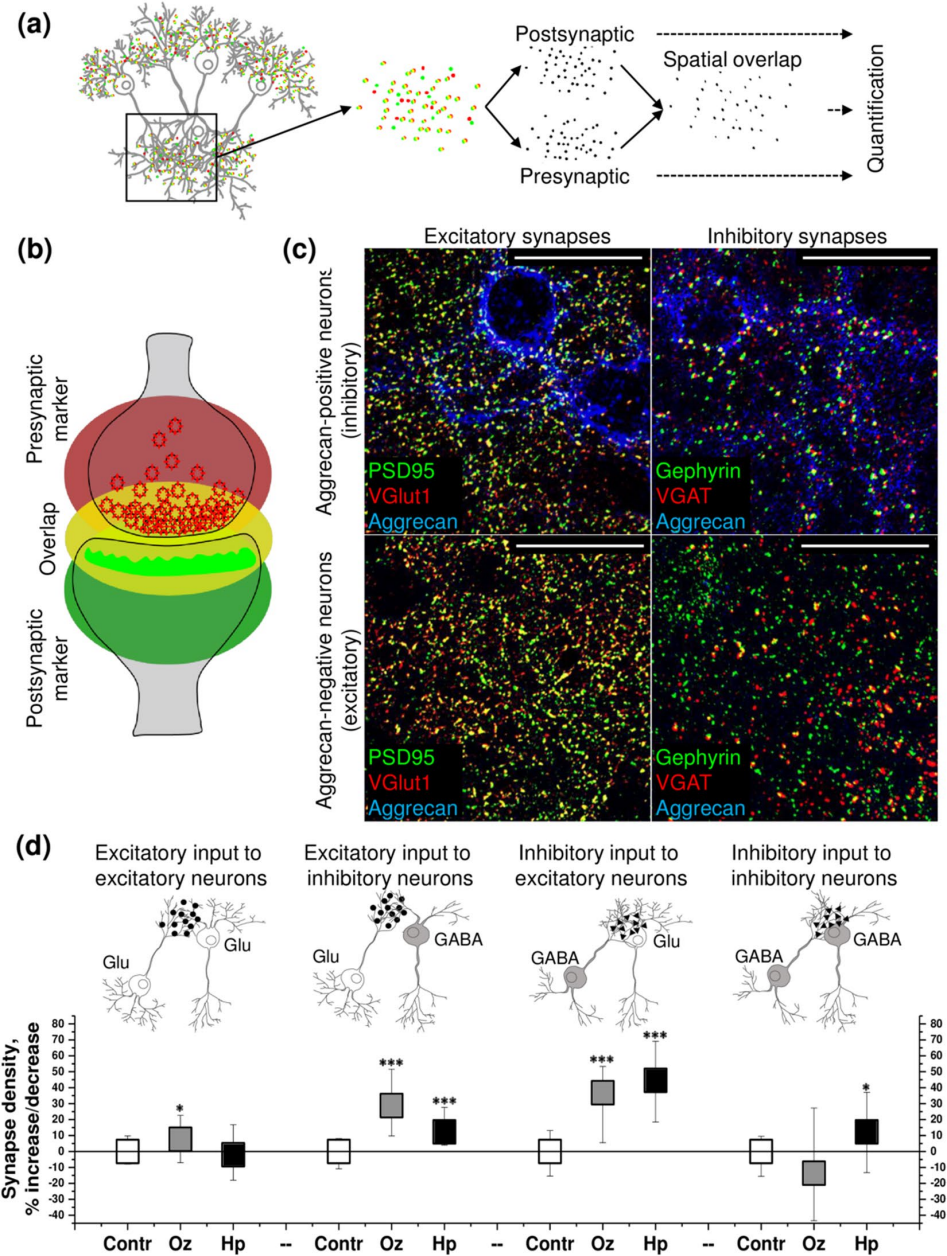
Olanzapine (Oz) and haloperidol (Hp) modify the density of excitatory and inhibitory synapses to excitatory and inhibitory neurons. (**a**) The principle of the synapse quantification method is based on the detection of presynaptic and postsynaptic markers. (**b**) The overlap between presynaptic and postsynaptic fluorescence allows to identify the structurally complete synapses. Neurotransmitter transporter proteins are labeled in red at the presynapse, scaffolding protein is marked green at the postsynapse. (**c**) Glutamatergic and GABAergic synapses are detected with reference to aggrecan expression in 21 days *in vitro* (21 DIV) neuronal cultures. The representative 66.5 × 66.5 µm single plane confocal micrographs exemplify the staining patterns of excitatory (colocalization of PSD95 and VGlut1) and inhibitory (colocalization of gephyrin and VGAT) synapses in proximity to inhibitory (aggrecan-positive) and excitatory (aggrecan-negative) neuron somata (See explanation in the text). High-resolution scans were obtained from the regions proximal to neuronal somata. Scale bar, 30 µm. (**d**) The percent of increase/decrease in synapse density upon treatment with antipsychotics is shown as median (square center), and the inter quartile range (25–75% IQR whiskers). Each data point reflects the quantification of minimum 35 images (66.5 × 66.5 µm area, containing a single cell body of a 21 DIV neuron, exemplified in (**c**), *N* = 5. The asterisks indicate significant differences with the corresponding control, based on the Kruskal-Wallis ANOVA test (*p < 0.05; ***p < 0.001).

The density of gabaergic and glutamatergic synapses was quantified in 66.5 × 66.5 µm^2^ areas, containing the single soma of either an inhibitory aggrecan-positive interneuron or an aggrecan-negative excitatory neuron. The spatial overlap between immunochemically labeled presynaptic and postsynaptic markers was scored as indicating structurally complete synapses (Fig. 1a,b). Thereby, the overlap between gephyrin and vesicular GABA transporter (VGAT) marked inhibitory synapses on excitatory and inhibitory neurons. Conversely, the co-localization of postsynaptic density protein 95 (PSD95) and vesicular glutamate transporter type 1 (VGlut1) staining revealed the excitatory inputs to excitatory and inhibitory neurons (Fig. 1c). Using this approach, it was possible to classify and quantify the densities of four connection types within the network (Fig. 1d). Thus, excitatory and inhibitory synapses could be separately quantified on both inhibitory and excitatory neurons.

Our data indicated that both olanzapine and haloperidol promoted the excitatory input to inhibitory interneurons (by 29% and 12% respectively, compared with the control), while only olanzapine increased the excitatory input to excitatory cells (by 8%, compared with the control). Further, both olanzapine and haloperidol favored the inhibitory input to excitatory neurons (by 37% and 45% respectively, compared with the control), while only haloperidol enhanced the inhibitory input to inhibitory cells (by 12%, compared with the control) (Fig. 1d).

### *In silico* simulation predicts the distinct patterns of network activity, induced by the antipsychotics

**Figure 2:**
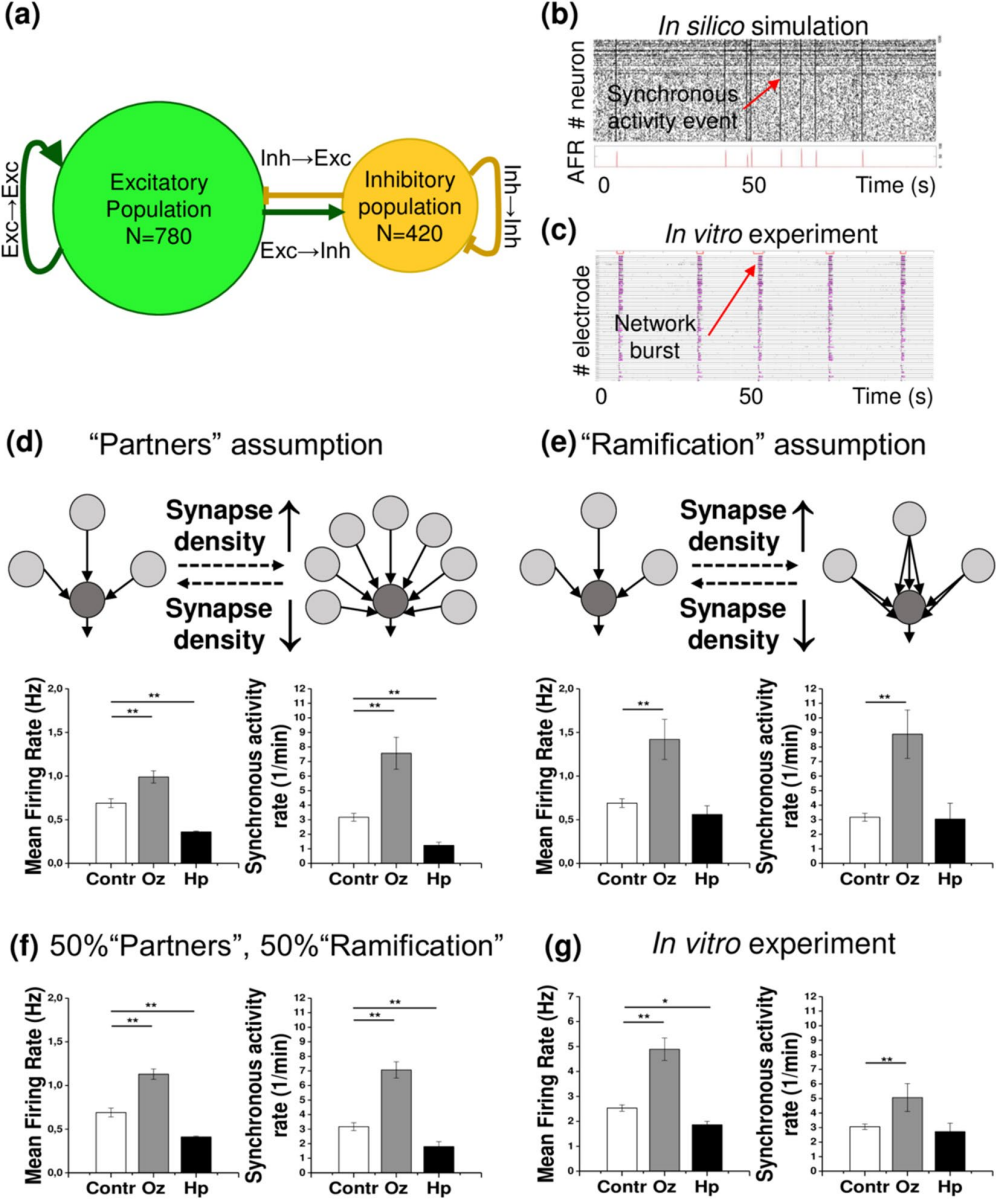
*In silico* simulations predict the neural network activity changes induced by olanzapine (Oz) and haloperidol (Hp) based on synapse density modifications. (**a**) The schematic illustrates the composition and connection types of the *in silico* networks. (**b**,**c**) Exemplary raster plots of *in vitro* and *in silico* network activity. (**b**) The raster plot of a 100 sec episode of simulated network activity (#1–780 excitatory and #781–1200 inhibitory neurons) illustrates the stationary activity pattern. The instantaneous average firing rate (AFR) is plotted to visualize the synchronous network activity events. (**c**) The raster plot of 100 sec episodes of activity in 60 MEA electrodes is shown for a control 21 DIV culture, exemplifying the representative network activity pattern. Black hatches indicate single spikes, magenta lines stand for bursts, red bars (the upper line in each stack) mark the network burst events. (**d**) The cartoon illustrates the “Partners” assumption for simulation. The quantifications below display mean firing and synchronous activity rates, predicted by the simulation. (**e**) The cartoon illustrates the “Ramification” assumption. The quantifications below display mean firing and synchronous activity rates, predicted by the simulation. (**f**) Mean firing and synchronous activity rates were quantified following the “Mixed” assumption simulation. In this experiment, it was assumed that 50% of synapse density modifications contribute to connectivity changes in accordance with the “Partners” assumption, and the other 50% with the “Ramification” assumption. Each bar represents the mean ± SEM value, *N* = 5. (**g**) Mean firing rate and synchronous activity rate (network burst frequency) of mature (21 DIV) *in vitro* networks are shown. Each bar represents the mean ± SEM value, *n* = 4. The asterisks indicate significant differences with the control, based on the Kruskal-Wallis ANOVA test (*p < 0.05; **p < 0.01; ***p < 0.001).

Given that both olanzapine and haloperidol modified synapse density, we further asked whether the observed network connectivity changes could result in distinguishable differences in activity patterns. To answer this question, we constructed an *in silico* neural network which closely resembles the *in vitro* neuronal culture (see Methods). All pharmacological treatments were mimicked as connectivity parameter changes, mirroring the detected synapse density modifications (Fig. 2a). For all experimental conditions, the simulations closely replicated the spontaneous activity patterns of neuronal cultures *in vitro*, examined with multiple electrode arrays (MEAs, see below). This is reflected in exemplary raster plots comparing *in silico* and *in vitro* network activity (Fig. 2b,c). Notably, the simulated networks displayed short episodes of synchronized activity when all neurons were spiking coherently. We suggest that these synchronous activity events correspond to the known network burst phenomena^30^.

To understand the links between synapse density, network connectivity and neural activity, we assumed two different modes of how synapse density may relate to network connectivity: “Partners” and “Ramification” assumptions, illustrated in (Fig. 2d,e). The increase or decrease of the amount of inputs to a particular cell can depend on i) the changes of the number of partners, providing the inputs (“Partners” assumption), and ii) the changes in the multiplicity of a single neuron-to-neuron connection (“Ramification” assumption). In real networks, these two modes are probably combined, although they may potentially account for different activity changes. For this reason, we separated the two assumptions in our simulations.

Under the “olanzapine” condition mean firing rates and the frequency of synchronized activity events were increased in both scenarios. In contrast, when the impact of haloperidol treatment on synaptic connectivity was implemented in the simulation, the “Partners” assumption led to a decreased mean firing rate and frequency of synchronized activity events, while no significant differences were observed under the “Ramification” assumption.

Therefore, it appears that both assumptions partially reproduced the results of the *in vitro* network activity measurements. To mimic the effects of the antipsychotics more precisely in the *in silico* system, the “Mixed” assumption was tested (Fig. 2f). The “Mixed” assumption proposes that half of the synapse density modifications is translated into network connectivity changes in accordance with the “Partners” assumption whereas the residual half mirrors the “Ramification” mode. When the three simulation concepts were compared, the “Mixed” assumption most faithfully predicted the *in vitro* network activity changes recorded experimentally (Table 1).

**Table 1:** Olanzapine (Oz) and haloperidol (Hp) modify network activity in both *in silico* and *in vitro* models.

Increase/decrease (% of control)		*In silico* simulation			*In vitro* Network activity
		“Partners” assumption	“Ramification” assumption	“Mixed” assumption	
Mean firing rate	Oz	44 ± 9%	107 ± 33%	65 ± 9%	93 ± 9%
	Hp	−48 ± 2%	≈	−40 ± 2%	−26 ± 2%
Synchronous activity rate	Oz	139 ± 35%	180 ± 52%	123 ± 18%	65 ± 12%
	Hp	−61 ± 7%	≈	−43 ± 11%	≈

The changes of synchronous activity and mean firing rate upon treatments are shown as mean ± SEM difference with the control value (in % of the control value). Four independent experiments were assessed (*N* = 4). Only statistically significant values are indicated (p < 0.05, based on the Kruskal-Wallis ANOVA test). Abbreviations, Oz: olanzapine; Hp: haloperidol.

### Olanzapine and haloperidol modify spontaneous network activity

**Figure 3:**
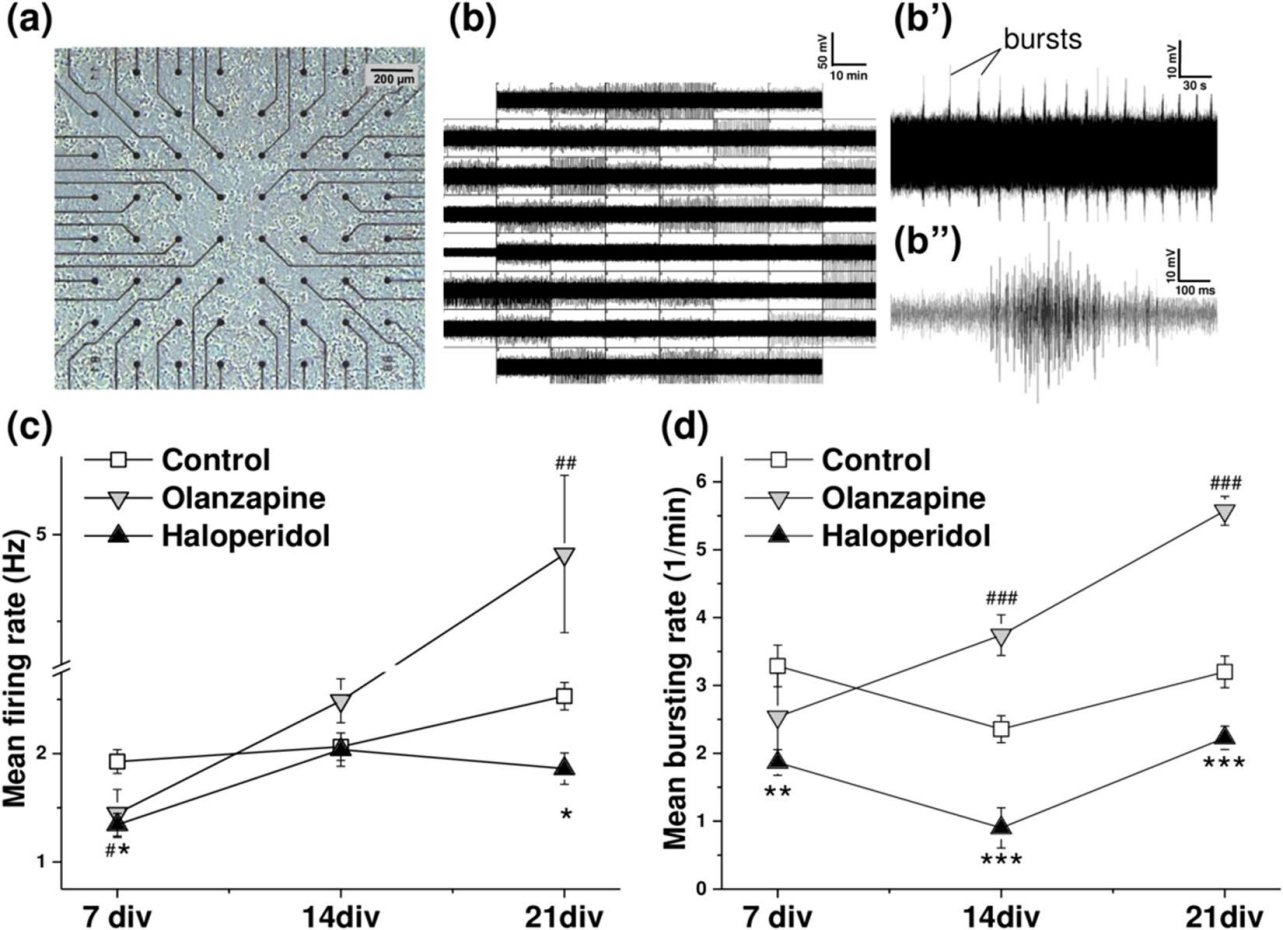
Olanzapine and haloperidol modify spontaneous activity at different stages of network maturation. (**a**) Neuronal culture (21 days *in vitro*) grown on a multiple electrode array (MEA). (**b**(**b”**)) Electrical activity of mature (21 days *in vitro*) neuronal cultures. (**b**) The panel exemplifies a 15 min period of neural activity, recorded in 59 channels. The layout is arranged in accordance with the electrode position, shown in (**a**). (**b’**) The regular bursting pattern of activity detected by a single electrode. (**b”**) The line graph exemplifies a single burst structure. (**c**,**d**) White squares represent the control condition, grey downward triangles stand for the olanzapine and upward black triangles for the haloperidol treatment condition. The asterisks and hashes indicate significant differences with the control at a particular stage of maturation (haloperidol and olanzapine conditions respectively), based on the Kruskal-Wallis ANOVA test (*^,#^p < 0.05; **^,##^p < 0.01; ***^,###^p < 0.001). (**c**) Quantification of mean firing rate after 7, 14 and 21 days *in vitro* (DIV). Each data point indicates the mean ± SEM (Hz) of minimum 159 active electrodes, *N* = 4 (15 min recording time). (**d**) Quantification of mean bursting rate at 7 DIV, 14 DIV and 21 DIV stages of maturation. Each data point displays the mean ± SEM (bursts per minute) of minimum 41 (at 7 DIV), 48 (at 14 DIV) and 119 (at 21 DIV) bursting electrodes, *N* = 4 (15 min recording time).

To investigate the effects of olanzapine and haloperidol on spontaneous network activity in neuronal cultures, recordings were performed using multiple electrode arrays (MEAs). We used square 8 × 8 electrode (center-to-center distance 200 µm) arrays to monitor the network activity changes over 21 days of cultivation (Fig. 3a). Each electrode locally senses the electrical potential in a radius of 30 µm from its center, with high temporal precision^31^. Thus, the recorded “spikes” represent a group response from several (typically 5 to 10) neurons, located near to the electrode (Fig. 3b(b”)). In agreement with other investigators^32, 33^, we will further designate such groups of neurons as “units”.

In our approach, the antipsychotic treatments were chronically applied on day 3, 11 and 19 of cultivation. At the early stage of 7 days *in vitro* (DIV), both drugs slightly, but significantly decreased the mean firing rate (Fig. 3c). After 14 days, the mean firing rate in treated cultures did not significantly differ from the control condition, but the mature networks at 21 DIV stage exhibited in the presence of haloperidol a reduced and upon exposure to olanzapine an enhanced firing rate.

The effects of antipsychotics on the bursting behavior of treated cultures also evolved as the networks maturated. Haloperidol decreased the bursting rate at all stages starting with 7 DIV, but the strongest effect was observed after 14 days of cultivation (Fig. 3d). Olanzapine gradually increased the bursting rate at the 14 DIV and 21 DIV stages, whilst the young 7 DIV cultures did not differ from the control condition.

As the cultivated neuronal networks maturated, their spontaneous activity became more synchronized. In our experiments, network bursts were prominent in 21 DIV cultures (Fig. 2c,g). Network burst rate quantification revealed that olanzapine treated cultures were synchronized at significantly higher frequency (5.1 ± 0.9 versus 3.1 ± 0.2 network bursts per minute, compared with the control condition).

### Olanzapine and haloperidol differentially affect the activity of excitatory and inhibitory units

**Figure 4:**
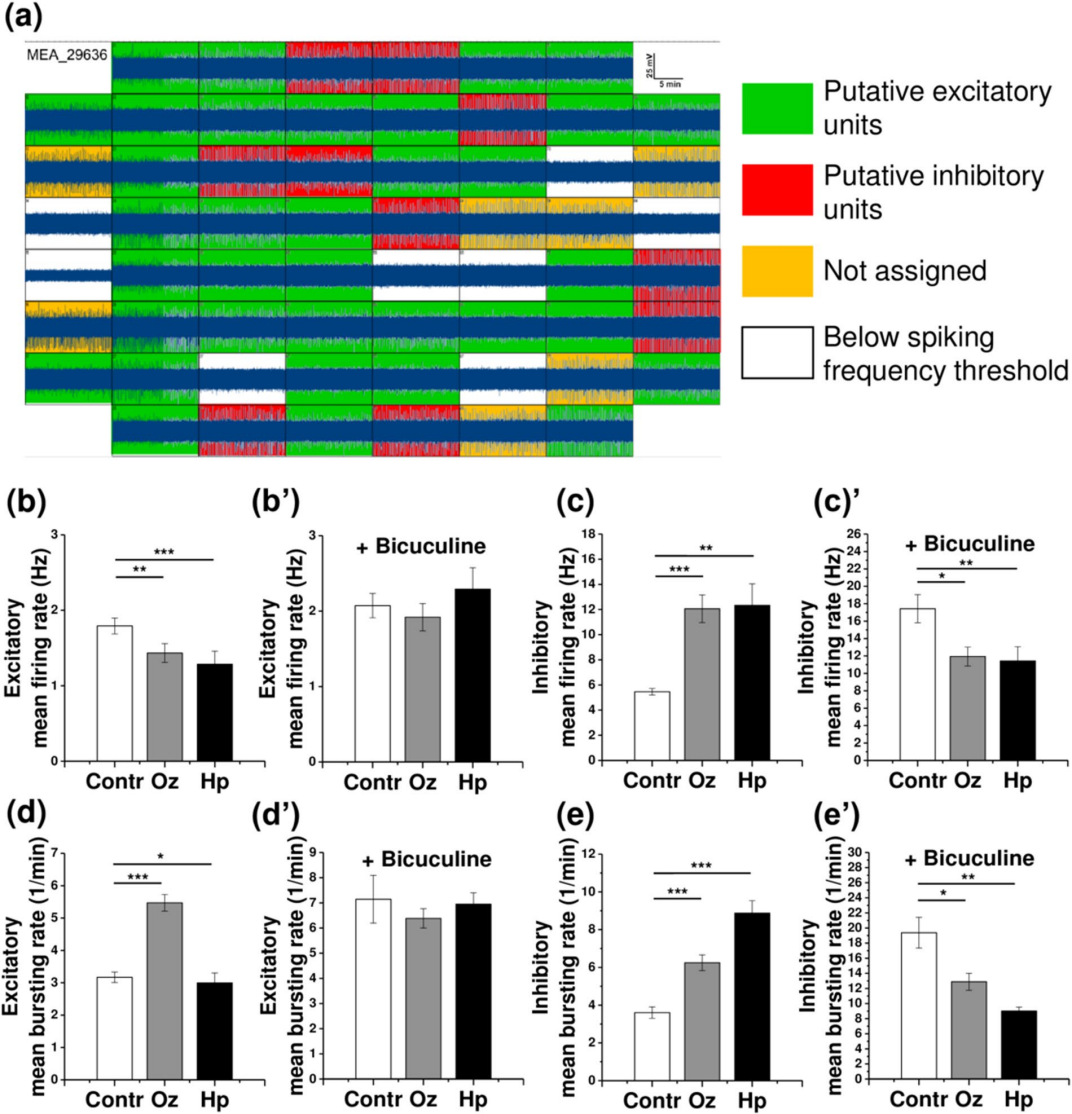
Individual analysis of inhibitory and excitatory units reveals differential effects of olanzapine (Oz) and haloperidol (Hp) on the spontaneous activity of putative inhibitory and excitatory populations of neurons. (**a**) Excitatory and inhibitory unit assignment. The 8 × 8 layout of a MEA chip demonstrates a representative 15-minute recording episode (high pass filtered data is shown). The active electrodes (above the 0.2 Hz spiking frequency threshold) are sorted into putative excitatory and inhibitory units, based on hierarchical cluster analysis (see Methods). (**b**–**e’**) Network activity quantifications are shown for 21 days *in vitro* cultures. (**b**) Mean firing rate of excitatory units (minimum 115 electrodes, *N* = 4). (**b’**) Mean firing rate of excitatory units after bicuculline application, same electrodes as in (**b**). (**c**) Mean firing rate of inhibitory units (minimum 32 electrodes, *N* = 4). (**c’**) The effects of treatments are reversed after bicuculline application, same electrodes as in (**c**). (**d**) Mean bursting rate of excitatory units (minimum 115 electrodes, *N* = 4). (**d’**) Mean bursting rate of excitatory units after bicuculline application, same electrodes as in (**d**). (**e**) Mean bursting rate of inhibitory units (minimum 32 electrodes, *N* = 4). (**e’**) The effects of treatments are reversed after bicuculline application, same electrodes as in (**e**). The asterisks indicate significant differences with the control, based on the Kruskal-Wallis ANOVA test (*p < 0.05; **p < 0.01; ***p < 0.001).

Since olanzapine and haloperidol differentially affected GABA- and glutamatergic synapse density at excitatory and inhibitory neurons, we expected that the activity of excitatory and inhibitory units of the cultivated networks should also be modified accordingly. To examine this possibility, we classified the active MEA electrodes into putative excitatory and inhibitory units. The units were assigned to either category based on the cluster analysis of single electrode spiking parameters (Fig. 4a). A similar classification approach has previously been applied for distinguishing between inhibitory and excitatory units and confirmed by the morphological identification of inhibitory neurons^33^.

The activity of excitatory and inhibitory units was differentially modified by exposure to the antipsychotics, in agreement with the modification of inhibitory or excitatory synaptic innervation reported above (Fig. 2d). Both olanzapine and haloperidol decreased the mean firing rate (MFR) of excitatory units (Fig. 4b). The application of bicuculline methiodide, a well-known inhibitor of GABAergic transmission abolished the differences of MFR between the different treatment groups (Fig. 4b’). The MFR of inhibitory units was intensified upon the treatment with both antipsychotics (Fig. 4c). This effect was reversed by bicuculline application, as expected for GABAergic neurotransmission (Fig. 4c’).

The mean bursting rate (MBR) was also altered in inhibitory and excitatory neuron populations. Compared with the control, olanzapine treatment significantly increased both the bursting rate of excitatory and inhibitory units (Fig. 4d,e). These effects were reversed after bicuculline application (Fig. 4d’,e’). Haloperidol slightly decreased the MBR of excitatory and enhanced the MBR of inhibitory units, where both effects were sensitive to bicuculline application (Fig. 4d’,e’). In conclusion, the impact of antipsychotic treatments on burst frequency appeared to depend on GABAergic neurotransmission.

### Olanzapine and haloperidol modify the burst structure in excitatory and inhibitory units

**Figure 5:**
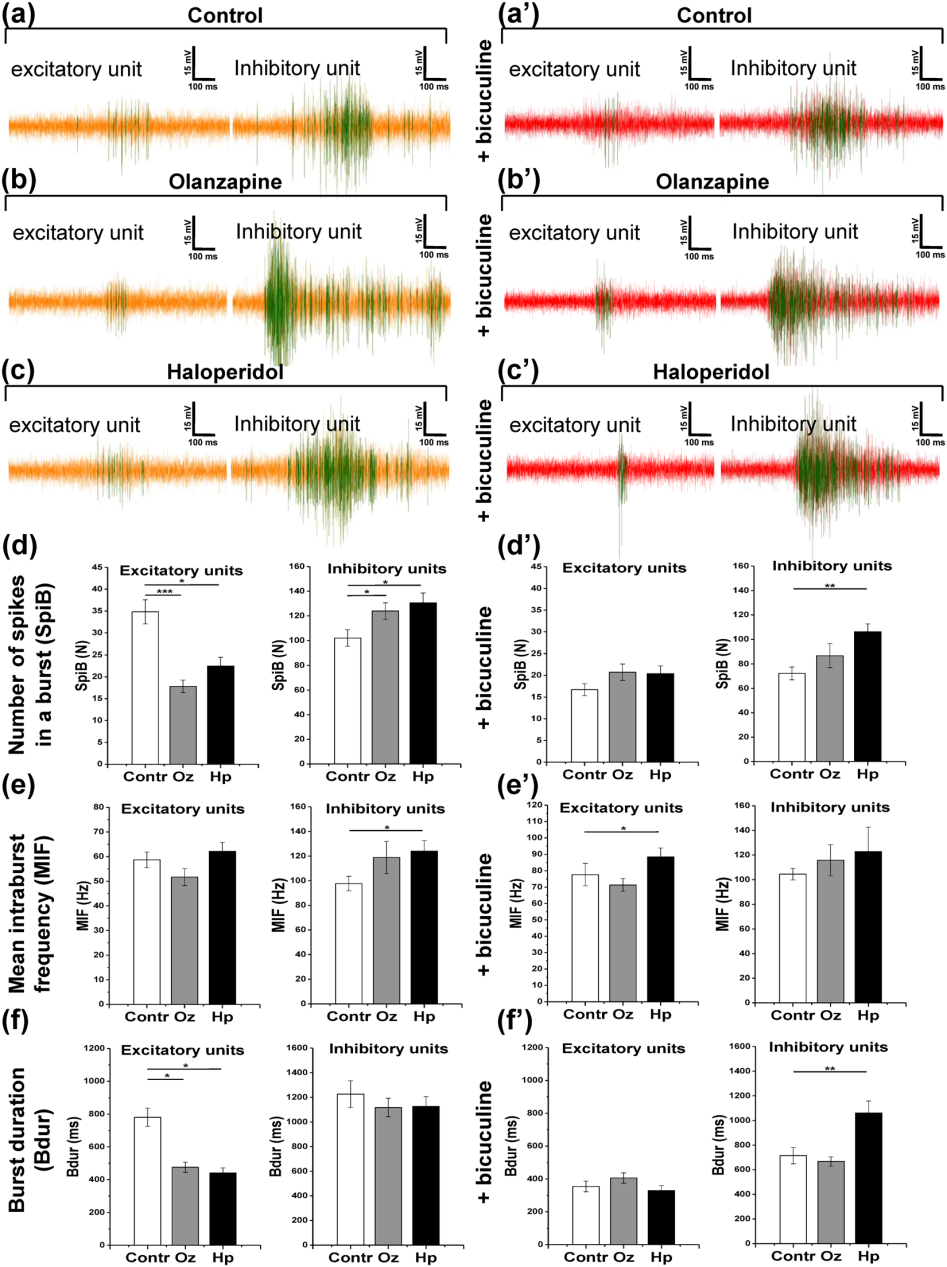
Olanzapine (Oz) and haloperidol (Hp) differentially modify burst structure in excitatory and inhibitory units. The activity line plots and quantifications are shown for 21 days in *in vitro* cultures. (**a**–**c**) The representative bursts are shown for putative excitatory and inhibitory units in control, olanzapine, and haloperidol conditions, respectively. (**a’**–**c’**) The bursts in the same units as in (**a**–**c**) are shown after 30 minutes of 6 µM bicuculline methiodide application. Vertical bar, 15 mV; horizontal bar, 100 ms. (**d**–**f**) The quantification of key burst structure parameters is shown for putative excitatory units (minimum 115 electrodes, *N* = 4) and inhibitory units (minimum 32 electrodes, *N* = 4): number of spikes in a burst (SpiB) (**d**) mean intraburst spiking frequency (MIF) (**e**) and burst duration (Bdur) (**f**). (**d’**–**f’**) Burst structure parameters are quantified after 30 minutes of 6 µM bicuculline methiodide application, same units as in (**d**–**f**). The asterisks indicate significant differences with the control, based on the Kruskal-Wallis ANOVA test (*p < 0.05; **p < 0.01; ***p < 0.001).

To obtain a deeper understanding about how the antipsychotics affect excitatory and inhibitory neurons, we further analyzed the key parameters of burst structure in putative excitatory and inhibitory units: the number of spikes in a burst (SpiB), the mean intraburst spiking frequency (MIF), and the duration of bursts (Bdur). The representative line graphs of individual bursts are shown in (Fig. 5a–c).

As can be derived from the quantification of burst parameters both olanzapine and haloperidol increased the duration and number of spikes in excitatory bursts, while the mean intraburst frequency was not affected (Fig. 5d–f). The application of bicuculline masked the effects of pharmacological treatments in that it rendered the excitatory bursts in all groups more compact. Concomitantly, it increased the mean intraburst frequency and reduced the duration of bursts (Fig. 5a’–f’).

In inhibitory units, olanzapine slightly augmented the number of spikes in a burst, and haloperidol elevated the number of spikes and the mean intraburst frequency (Fig. 5d–f). Interestingly, bicuculline application reduced the duration of bursts and the number of spikes in a burst without changing the mean intraburst frequency in control and olanzapine conditions. Bicuculline did not, however, interfere with the inhibitory bursts in the haloperidol treated cultures (Fig. 5a’–f). Notably, the inhibitory bursts were generally longer and displayed higher intraburst frequency than the excitatory ones. Since bursting and high frequency spiking is characteristic for the inhibitory interneurons^34, 35^, the burst structure analysis supports the classification of units based on hierarchical clustering used in our analysis.

## Discussion

In previous studies, various antipsychotics have been shown to alter dendritic outgrowth, dendritic spine formation and synaptic proteins expression^10, 12, 36^. Recently, antipsychotic-induced alterations of synapse number were linked to network activity changes^11^. In order to understand the relations between the remodeling of local circuitry and network activity upon SGA and FGA treatments, we designed an interdisciplinary approach combining synapse quantification, computational modeling and spontaneous activity recording. In agreement with earlier reports, our data demonstrates that olanzapine and haloperidol modify both GABAergic and glutamatergic connectivity in cultured neural networks. In addition, both treatments increased inhibitory and attenuated excitatory firing, leading to distinct patterns of network synchronization. However, solely olanzapine treatment promoted excitatory input to excitatory cells, resulting in the increased bursting of excitatory units and augmented frequency of network bursts. Regarding psychosis, this effect may underlie better cognitive improvements under SGA treatments, compared to FGA medications^37^. The activity shifts observed in our *in silico* network model were driven by exclusively adjusting synapse number. Yet the resulting virtual faithfully replicated the *in vitro* activity patterns. This supports our interpretation that the functional effects of antipsychotics detected in our study are most probably caused by rearrangements of network connectivity.

In the intact brain, the influences of antipsychotics on the dopaminergic, serotonergic, GABAergic and glutamatergic signaling converge, resulting in a synergistic effect on both large-scale neural networks and local circuitry. To explicitly investigate the effects of olanzapine and haloperidol on inhibitory and excitatory innervation, we used pure primary cultures of hippocampal neurons that lack serotonergic, dopaminergic and cholinergic inputs from other brain regions. Despite the afferent innervation was missing, dopamine and serotonin receptors are known to be involved in the regulation of synaptogenesis in analogous *in vitro* models. For example, a recent study^36^ demonstrated that the activation of dopamine D2 receptors increases the dendritic spine density, mediated by the extracellular signal-regulated kinase (ERK) pathway.

In the employed *in vitro* model, the dissociated neurons settle onto the substrate randomly, and the initial connectivity pattern is likely established through coincidental membrane contacts between the sprouting neurites. In the computational model that we used, this process is mirrored by the random connectivity in the *in silico* network. Notably, this mode of synaptic connectivity establishment has proven physiologically relevant^38^. In our computational model, all available macroscopic parameters were translated from the *in vitro* cultures to the *in silico* networks. These included average synapse numbers, the total number of neurons, and the proportion of inhibitory interneurons. This procedure resulted in the close replication of the *in vitro* network activity, which underscores the validity of our modeling.

Interneurons provide the control of excitability and synchronization in neural networks and are crucial for maintenance of functional states. Interneurons are diverse with respect to their morphology, molecular profiles, functions and target regions^21, 34^. Interestingly, subpopulations of interneurons can be distinguished based on the expression of the calcium-binding proteins parvalbumin, calretinin and calbindin^21^. In mature human and rodent brains, the somata and proximal dendrites of interneurons containing parvalbumin are coated with a complex layer of extracellular matrix constituents, the so-called perineuronal net (PNN)^20, 39^. Although in the adult mouse brain a subpopulation of excitatory neurons in cortex and hippocampus can also express PNNs, in our neuronal cultures more than 95% of the parvalbumin positive neurons also expressed aggrecan, a major PNN component^40^, suggesting that primarily the inhibitory interneurons carried PNNs in our model. Thus, we could use aggrecan as a marker of interneurons, and to define the average density of GABA- and glutamatergic inputs, received by excitatory and inhibitory neurons. Applying this strategy, network connectivity remodeling could be detected following the treatment with antipsychotics.

The number of synapses connecting the cell body determines the input strength to an individual neuron. Synapse density may be regulated in different ways. For example augmented synapse number could result from a larger number of connecting partner cells. Alternatively, an individual connecting partner cell may expand the number of postsynaptic neurotransmitter release sites, thereby raising postsynaptic currents. Although the majority of patch-clamp-based neuron to neuron signaling paradigms suggest monosynaptic transmission, studies of neuronal microcircuits clearly demonstrated that two partner neurons can form multiple synapses^41, 42^. Moreover, recent studies have concluded a low probability for the establishment of monosynaptic connections within several types of neuronal networks^43^. The formation of functional multi-synaptic connections seems to underlie rewiring and plasticity of neocortical microcircuits during development^44^. Taken together, the number of synapses found at a particular neuron most likely depends on both multiplicity of one-to-one contacts and the number of different connecting partners. Using the immunocytochemistry-based method presented in this study we were unable to discriminate between these two possible sources of synapses. However, we attempted to separate them in network simulations by introducing the effects of antipsychotic treatments as the changes in the number of cells that wire together (“Partners” assumption), and as the single input strength modification (“Ramification” assumption). The simulation using the combination of both modes in the so-called “Mixed” assumption most accurately replicated the experimental data collected by MEA-recordings. In conclusion, our observations support the interpretation that olanzapine and haloperidol modify both the number of synapses generated by an individual neuron and the number of connecting neuronal partners in our cultured neuron network.

On the cell biological level, the plasticity of connectivity may involve parameters such as neurite sprouting and outgrowth, and the formation of dendritic spines. Alternatively, neurons might modify synapse density by developing autapses, synapses established on the own cell body. Autapses are generated *in vitro* ^45^, but they cannot be discriminated by the immunocytochemistry based synapse quantification that we performed. This drawback of the method may curtail the applicability to the *in vivo* situation. In our computational model, the autaptic connections were allowed, but were not considered further.

Independent from the changes concerning the number of synapses that we observed, antipsychotics can modulate neurotransmission at the level of individual synapses^46, 47^. In our simulations, the “application” of olanzapine and haloperidol treatments was modeled based on synapse density quantification. Notably, the adjustment of connectivity parameters alone was sufficient to closely replicate the activity pattern changes that the drugs induced *in vitro*. In other words, the computational model predicted that if the antipsychotics would affect nothing but synapse formation in neuronal cultures, the network activity would be modulated as detected in the MEA recordings.

Considering *in vitro* network activity, the separate analysis of excitatory and inhibitory units revealed that their spiking and bursting behavior correlated closely with the synapse density modification, caused by olanzapine and haloperidol treatment. The activity patterns generated by distinct treatments were abolished by the application of bicuculline, highlighting the importance of GABAergic transmission in the network. Indeed, the consequences of antipsychotic treatments on inhibitory units were even reversed upon GABA receptor blockade. This observation was surprising because the extent of excitatory inputs to inhibitory neurons was increased by both pharmacological treatments. The putative disinhibition of glutamatergic cells by bicuculline should hence have escalated the activity of inhibitory interneurons. An explanation of this discrepancy may be derived from the burst structure analysis.

Under control conditions, the excitatory bursts were almost twice longer than in the treatment situation. Since bursts repeatedly stimulate the interneurons over a long culture period they may potentiate excitatory synapses on inhibitory cells. Analogous to the higher amplitude of long-term potentiation induced by high frequency stimulation^48^, the excitatory synapses might be more strongly potentiated in the absence of treatment. Under these circumstances antagonizing the GABA signaling with bicuculline may yield a stronger excitatory input to the inhibitory neurons. While this hypothesis provides an appealing explanation for our findings, the analysis of synaptic strength in our model remains to be performed in future investigations.

In conclusion, our data suggests that both olanzapine and haloperidol may contribute to restore a compromised excitation-inhibition balance in neuronal networks. In light of the recently proposed theory that glutamate based excitatory signaling is implicated in the pathophysiology of psychosis (for reviews see refs 5 and 49), our study paves a way to explore how antipsychotic drugs modify local neuronal circuitry.

## Methods

### Animal housing and ethical standards

The present study was carried out in accordance with the European Council Directive of September 22, 2010 (2010/63/EU) for care of laboratory animals and approved by the animal care committee of North Rhine-Westphalia, Germany, based at the LANUV (Landesamt für Umweltschutz, Naturschutz und Verbraucherschutz, Nordrhein-Westphalen, D-45659 Recklinghausen, Germany). The study was supervised by the animal welfare commissioner of Ruhr-University. Male and female NMRI mice were housed individually with a constant 12-h light-dark cycle and access to food and water *ad libitum*. All efforts were made to reduce the number of animals in the experiments.

### Cell cultures

Indirect cocultures of neurons and astrocytes were obtained and maintained as described previously^25, 50, 51^. In brief, the hippocampi were obtained from the embryos of wild type NMRI mice at 15, 5 days post conception, gently dissociated and plated out to achieve ≈20000 cells/cm^2^ density. Starting the first day in culture, the neurons were supplemented with astrocyte monolayers, using the cell culture inserts (BD Falcon #353095).

### Immunocytochemical procedures

After 21 days in culture, neurons were fixed with 4% w/v paraformaldehyde (Sigma-Aldrich) for 10 min at room temperature and processed as described previously^52^. For synapse detection, we used the antibodies against vesicular glutamate transporter VGLUT1 (guinea pig anti-VGLUT1, 1:500, Synaptic Systems, #135304), postsynaptic density protein PSD95 (mouse anti-PSD95, 1:500, Millipore, MAB1598), vesicular GABA transporter VGAT (guinea pig anti-VGAT, 1:500, Synaptic Systems, #131103), and gephyrin (mouse anti-gephyrin, 1:500, Synaptic Systems, #147011). For cell type identification, the antibodies against GABA (rabbit anti-GABA, 1:2000, Sigma-Aldrich, A2052), parvalbumin (chicken anti-parvalbumin, 1:500, Synaptic Systems, #195006) and aggrecan (rabbit anti-aggrecan, 1:500, Millipore, AB1031; mouse anti-aggrecan, 1:500, R&D Systems, MAB1220) were applied. To enable the immunofluorescence detection, the following secondary antibodies were used: anti-mouse IgG Alexa 488 (1:250, Jackson Immuno, #715-545-150), anti-mouse IgG Alexa 594 (1:500, Jackson Immuno, #715-545-151), anti-mouse IgG Alexa 647 (1:500, Jackson Immuno, #115-605-003), anti-rabbit IgG Alexa 594 (1:500, Jackson Immuno, #711-585-152), anti-guinea pig IgG Alexa 647 (1:500, Jackson Immuno, #706-605-148), and anti-chicken IgY Alexa 488 (1:250, Jackson Immuno, #103-545-155).

### Synapse density quantification

Synapse density quantifications were performed with the self-developed plugin for ImageJ^53^ software as described previously^52^. Briefly, the structurally complete GABAergic and glutamatergic synapses were identified by the spatial overlap of pre- and postsynaptic markers (VGAT-gephyrin and Vglut1-PSD95 and were quantified in 66.5 × 66.5 µm^2^ surface areas, containing a single neuron body, based on the spatial overlap between the presynaptic and the postsynaptic marker staining. As far as the majority of inhibitory synapses are established at the soma, and excitatory synapses - at the dendrites and soma^34, 54^, we suggest that our synapse quantification reflects the input, which is received by a particular cell from its network partners.

In brief, the structurally accomplished GABAergic and glutamatergic synapses were identified by the spatial overlap between presynaptic and postsynaptic markers^52^. The synapse density was then analyzed with our in-house Synapse Counter plugin for ImageJ (freely available at https://github.com/SynPuCo/SynapseCounter).

### Network activity simulations

To investigate the potential functional outcome of synapse density modifications, induced by the antipsychotic treatments, we performed attractor network simulations, based on the spiking neuron model^35, 55^. In order to be able to apply our simulation results to the *in vitro* model we use, the *in silico* networks were designed on the basis of our experimental results. The number of neurons comprising the simulated network (1200) approximated the number of cells grown over the MEA electrode field (0.16 × 0.16 × 50000 = 1280). The network contained 420 inhibitory and 780 excitatory neurons, corresponding to the proportion of inhibitory interneurons in cultivated networks. These amounted to ≈35%, based on immunocytochemistry data (see Results). The spiking neuron model parameters were set according to a published model^55^, resembling the activity of excitatory hippocampus neurons and fast spiking inhibitory interneurons. The computation of 15 minutes of activity was performed using 1 ms time steps. Every simulation experiment was repeated 5 times, with the connectivity matrix generated independently for each repetition, based on synapse density quantification data (see Supplementary Methods). Mean firing rate of a network was calculated by averaging the firing rates of all 1200 neurons. The synchronous activity rate was quantified as the average number of network-wide synchronous activity events per minute of simulation.

### Spontaneous network activity recording and analysis

Spontaneous activity of cultivated neuronal networks was measured using the cell culture compatible multiple electrode array (MEA). Square 8 × 8 electrode (center to center distance 200 µm) MEA chips (60MEA200/30iR-Ti, Multi Channel Systems) were used. In all experiments, spontaneous network activity was recorded for 15 minutes (temperature stabilized at 35 °C, gas exchange prevented) with the sampling frequency of 20000 Hz, using MC Rack software and the MEA1060 amplifier. The obtained data was analyzed in MatLab using the SpyCode toolbox for MEA analysis^56^, kindly provided by Dr. Michela Chiappalone (see Supplementary Methods).

### Cluster analysis of spontaneous activity and unit classification

To identify the excitatory and inhibitory units, all active electrodes of a MEA chip were classified into two cohorts. For each electrode, the mean firing rate (MFR), firing rate variance (Var) and firing rate Fano Factor (FF = Var/MFR) were computed using the SpyCode toolbox. Using these three variables, hierarchical cluster analysis (k = 2) was performed for each MEA separately using OriginPro2016G software. Classification was repeated with three methods: Ward, Group Average and Nearest Neighbor. When the activity of a single unit was clearly standing out during visual inspection (based on Euclidean distance) and separated from the clusters, the electrode was excluded from the analysis. Units, which were not equally assigned by the three classification methods, were defined as “not assigned” and were excluded from the analysis. A similar unit classification approach has previously been applied to precisely identify inhibitory and excitatory units in an analogous *in vitro* system, and was confirmed by the morphological identification of inhibitory neurons^33^.

### Pharmacological treatments

To model the chronic administration of antipsychotics in patients, 100 nM olanzapine (Eli Lilly and Co) and 100 nM haloperidol (Janssen) were added on day 3, 11 and 19 of cultivation, dissolved in minimal Eagle’s medium (MEM). At these time points, half of the cultivation medium was replaced with fresh medium containing the double concentration of the drug. In the control condition, an equal volume of pure MEM was added. The continuous replenishment of the drug pool aimed to compensate for the activity dependent usage^47^ and allowed us to examine their effects during the early, premature and mature stages of neural network development in our setup^25, 50, 51^. The final concentrations of drugs were selected on the basis of the target concentration range of antipsychotics in patients’ blood plasma^29^, the target receptor affinity, and preliminary survival tests (see Supplementary Fig. S1d). To reveal the contribution of GABAergic signaling, 6 µM bicuculline methiodide (Sigma-Aldrich, #14343) was bath applied for 30 minutes prior to measurement.

### Statistics

To identify the composition of cultivated networks, 15 scanning areas (460.68 × 460.68 µm), obtained in 5 independent experiments, were analyzed per condition. For synapse density quantifications, at least 35 images (n = 35, 66.5 × 66.5 µm), obtained in 5 independent experiments (N = 5), were analyzed per condition. For spontaneous activity investigation, the data from 4 independent experiments (N = 4, 1 MEA per condition per experiment) was analyzed. In all figure legends, the number of independent experiments (biological replicates) is indicated as *N*. In network simulation experiments, 5 simulations were performed for each condition. The differences between groups were identified using the pairwise nonparametric Kruskal-Wallis ANOVA test (*p < 0.05, **p < 0.01 and ***p < 0.001 significance levels) in the OriginPro2016G software. For multiple corrections, the p values were manually adjusted using the Bonferroni method.

### Data Availability

All data generated or analysed during this study are included in this published article (and its Supplementary Information files).

## Electronic supplementary material

Supplementary information
