# Supplementary material for: The antipsychotic drugs olanzapine and haloperidol modify network connectivity and spontaneous activity of neural networks in vitro
Source: Sci Rep. 2017 Sep 14;7:11609. doi: 10.1038/s41598-017-11944-0 (PMC5599625; doi:10.1038/s41598-017-11944-0)
Supplement: Supplementary file 1 — Supplementary information [file 41598_2017_11944_MOESM1_ESM.doc]

**Supplementary information**

Antipsychotic drugs olanzapine and haloperidol modify network connectivity and spontaneous activity of neural networks *in vitro*

*Egor Dzyubenko^1^, Georg Juckel^2^ and Andreas Faissner^1^*

^1^Department of Cell Morphology and Molecular Neurobiology, Faculty of Biology and Biotechnology, Ruhr University Bochum, Universitaetsstr. 150, Ruhr-University D-44801 Bochum Germany, building NDEF 05;

^2^LWL University Hospital Department of Psychiatry, Psychotherapy and Preventive Medicine Ruhr University Bochum;

**Supplementary Methods**

**Connectivity parameters of *in silico* networks**

The random connectivity coefficients were based on the synapse quantification and were separately defined for excitatory input to excitatory neurons (EEcon), excitatory input to inhibitory neurons (EIcon), inhibitory input to excitatory neurons (IEcon), and inhibitory input to inhibitory neurons (IIcon). For example, the “control” coefficients were EEcon=0.20; EIcon=0.15; IEcon=0.07; IIcon=0.15, meaning that each excitatory neuron receives input from 20% randomly selected excitatory cells (not excluding itself), each inhibitory neuron receives input from 15% randomly selected excitatory cells, etc. The connectivity matrix was separately generated for each experiment and was not modified throughout the simulation. Synaptic weights (coefficients which reflect the strength of an input) were introduced as constants for each connection type (SWee, SWei, SWie, SWii) and were not modified throughout the simulation. The effects of antipsychotics were modeled by changing the connectivity parameters accordingly to the obtained synapse density quantification results. For the “olanzapine” and “haloperidol” conditions the simulations were performed under two assumptions: i) the changes of synapse density are proportional to the changes in the number of network input partners ("Partners" assumption); ii) the changes of synapse density modify the input strength from a particular partner, by adjusting the ramification of pairwise connections ("Ramification" assumption). Under the "Partners" assumption, connectivity parameters EEcon, EIcon, IEcon and IIcon were modified proportionally to synapse density changes. Under the "Ramification" assumption, synaptic weights SWee, SWei, SWie, SWii were adjusted proportionally to the square root of synapse density changes. This relation is based on the link between the number of connections and synaptic strength within neural networks with stabilized balance of excitation and inhibition ^1^. The "Partners" and "Ramification" hypotheses were further combined into the “Mixed” assumption, assuming that each mode explains about 50% of the observed synapse density changes. The detailed account of connectivity coefficients is presented in (Supplementary Table S1). Bicuculline application was modeled by setting SWie=0, SWii=0.

**Supplementary Table S1. The parameters of *in silico* network activity simulation.**

| Synaptic density modification | | | *In silico* simulation parameters | | | | | | | | | | | |
| --- | --- | --- | --- | --- | --- | --- | --- | --- | --- | --- | --- | --- | --- | --- |
| 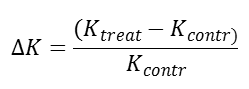 | | | Coefficient | | Control value | "Partners" assumption | | | "Ramification" assumption | | | “Mixed” 50/50 assumption | | |
| Input | Oz | Hp |  | | (*C*) | Relation | Oz | Hp | Relation | Oz | Hp | Relation | Oz | Hp |
| E→E | 0,08 | 0,00 | Connectivity  sparseness | EEcon | 0,20 | 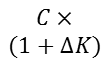 | 0,22 | 0,20 | 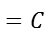 | 0,20 | 0,20 | 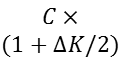 | 0,21 | 0,20 |
|  |  |  |  | EIcon | 0,15 |  | 0,19 | 0,17 |  | 0,15 | 0,15 |  | 0,17 | 0,16 |
| E→I | 0,29 | 0,12 |  | IEcon | 0,07 |  | 0,10 | 0,10 |  | 0,07 | 0,07 |  | 0,08 | 0,09 |
|  |  |  |  | IIcon | 0,15 |  | 0,15 | 0,17 |  | 0,15 | 0,15 |  | 0,15 | 0,16 |
| I→E | 0,37 | 0,45 | Synaptic  weight | SWee | 1,20 | 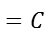 | 1,20 | 1,20 | 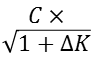 | 1,25 | 1,20 | 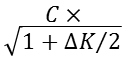 | 1,22 | 1,20 |
|  |  |  |  | SWei | 1,50 |  | 1,50 | 1,50 |  | 1,70 | 1,59 |  | 1,61 | 1,54 |
| I→I | 0,00 | 0,12 |  | SWie | -3,00 |  | -3,00 | -3,00 |  | -3,51 | -3,61 |  | -3,27 | -3,32 |
|  |  |  |  | SWii | -3,00 |  | -3,00 | -3,00 |  | -3,00 | -3,17 |  | -3,00 | -3,09 |

The coefficients that describe connectivity sparseness (EEcon, EIcon, IEcon, IIcon) and synaptic weights (SWee, SWei. SWie, SWii) are derived from the experimentally defined synaptic density modifications. *C*: control value; *K_treat_* and *K_contr_*: synapse density in treated and control conditions, respectively; E: excitatory; I: inhibitory; Oz: olanzapine; Hp: haloperidol.

**Multiple electrode array (MEA) data analysis**

The obtained data was analyzed in MatLab using the SpyCode toolbox for MEA analysis ^2^, kindly provided by Dr. Michela Chiappalone. A 200 Hz high pass Butterworth filter was applied to the raw data. Spike detection was performed using the Precision Timing Spike Detection (PTSD) algorithm (standard deviation coefficient = 8; peak lifetime period = 2 ms; refractory period = 1 ms; negative peak alignment). To prevent the contamination with the noisy data, only active electrodes (mean firing rate threshold was set to 0.2 Hz) were analyzed. The bursts were identified using the Burst Detection v2-PA algorithm ^2^, based on the adaptive search for the best threshold between the intra-burst and the inter-burst inter-spike intervals (ISI). The bursts were analyzed only for those units whose mean bursting rate (MBR) was higher than 0.4 bursts per minute. Network bursts were detected using the Burst Detection - PA algorithm ^2^, with the threshold of electrodes involved in a network burst set to 20%.

**References**

1 Barral, J. & D Reyes, A. Synaptic scaling rule preserves excitatory–inhibitory balance and salient neuronal network dynamics. *Nature neuroscience* **19**, 1690-1696, doi:10.1038/nn.4415 (2016).

2 Bologna, L. L. *et al.* Investigating neuronal activity by SPYCODE multi-channel data analyzer. *Neural Networks* **23**, 685-697, doi:10.1016/j.neunet.2010.05.002 (2010).


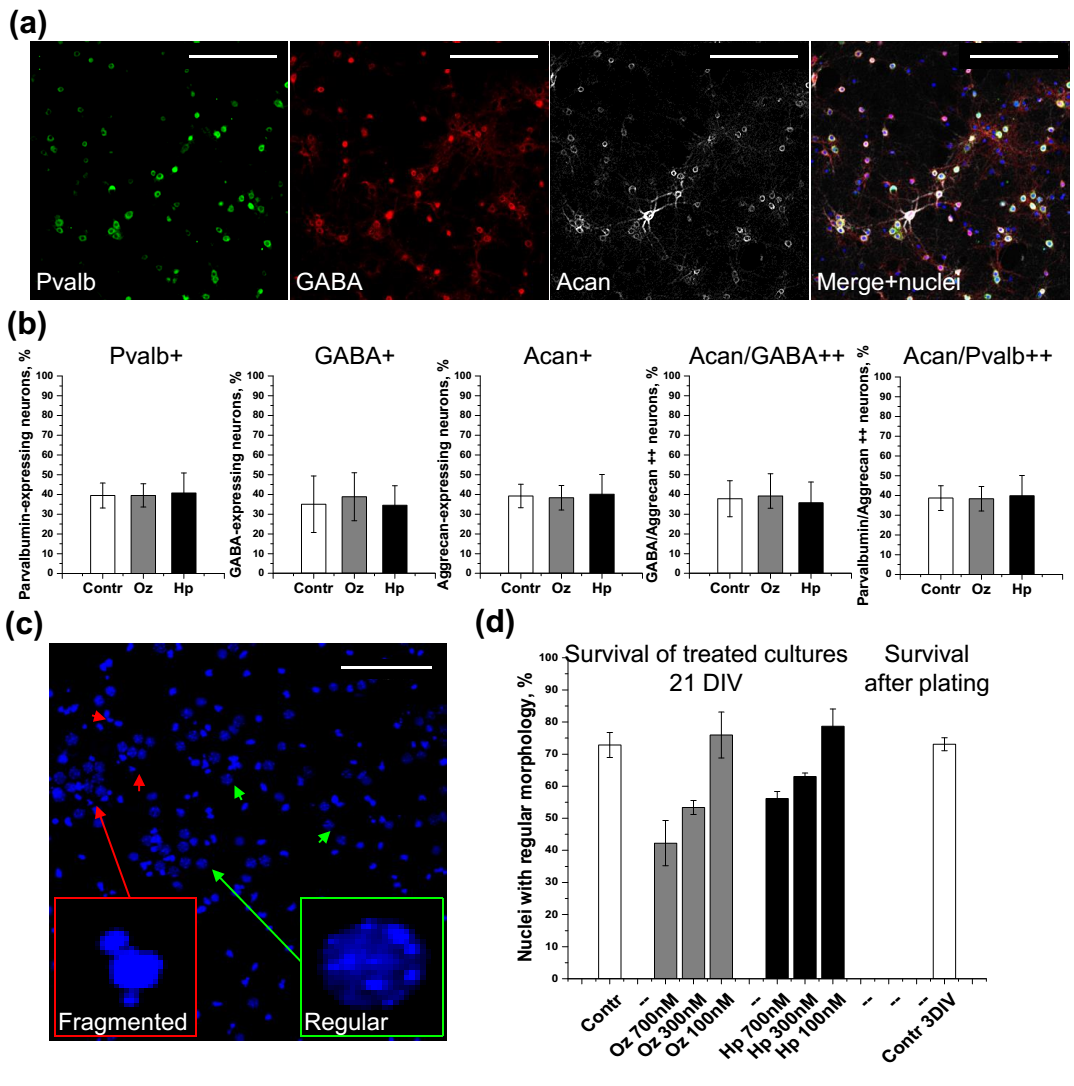


**Supplementary Figure S1. The composition of cultivated neuronal networks after 21 days in vitro is not affected by olanzapine (Oz) and haloperidol (Hp) treatments.**

**(a)**, The panel displays a representative immunocytochemical characterization of mature neuronal culture using key markers for interneurons: Parvalbumin (Pvalb), GABA and Aggrecan (Acan). The cell nuclei were stained with DAPI. A single plane low magnification confocal micrograph is shown. Scale bar, 200 µm. (**b)**, The proportion of inhibitory neurons is quantified in control, olanzapine and haloperidol treated cultures, based on the expression of parvalbumin, GABA, aggrecan, aggrecan-GABA colocalization and aggrecan-parvalbumin colocalization. The percentages indicate the number of positive cells, divided by the total number of nuclei. Each bar represents the mean ± SEM of 15 scanning areas (460.68x460.68 µm), N=5. **(c, d)**, Survival rate of cultivated neurons. The cell survival is estimated on basis of nuclei morphology. **(c)**, The morphology of neuronal nuclei (21 days in vitro neuronal culture) is revealed with DAPI staining. The nuclei with regular morphology are marked with green arrows (enlarged in green frame), apoptotic and necrotic nuclei – with red arrows (enlarged in the red frame). Scale bar, 100 µm. **(d)**, The quantifications of nuclear morphology demonstrate that the treatment with 300 nM and 700 nM Olanzapine (Oz) and Haloperidol (Hp) results in decreased neuron survival after 21 days in culture. In contrast, the survival in the presence of 100 nM olanzapine and haloperidol is not altered, compared with the control, and remains comparable to cell survival upon plating (control culture after 3 days in vitro – Contr 3 DIV). Each bar shows mean±SD for 12 imaging areas (460.68x460.68 µm), N=3.
